# Experiences of Care from Mental Health Services among Partners of Women Accessing Support for Postpartum Psychosis: a Qualitative Study

**DOI:** 10.1007/s10597-025-01479-4

**Published:** 2025-06-06

**Authors:** Emily Roxburgh, Billie Lever Taylor, Joanne Hodgekins

**Affiliations:** 1 Norwich Medical School, The University of East Anglia, Norwich, UK; 2 Division of Methodologies, Florence Nightingale Faculty of Nursing, Midwifery and Palliative Care, King’s College London, London, UK

**Keywords:** Postpartum psychosis, Services, Partners, Fathers, Qualitative, Postnatal care

## Abstract

Postpartum psychosis (PP) affects one to two per 1,000 women and requires urgent intervention. Whilst PP primarily impacts women, it can place significant strain on partners, who often play a pivotal role throughout postnatal care and recovery. Little is known about how mental health services engage with and support partners during a PP episode. Therefore, this study aims to explore partners’ experiences of mental health services to better understand their experiences and needs and inform service provision. Semi-structured interviews were conducted with 12 partners of women who received support for psychosis following childbirth. Findings were analysed thematically. Three main themes were identified: ‘understanding and responding to PP’, ‘partners’ involvement in PP care’ and ‘the responsibilities undertaken and partner wellbeing’. Partners struggled to recognise PP and reported variability in healthcare professionals’ knowledge of the condition. They urged for greater information and awareness of PP for partners, professionals and society. Most took on advocacy roles yet reported inconsistent involvement from services. Their ongoing responsibilities and PP related experiences significantly impacted their emotional wellbeing, with many finding services unresponsive to their needs. Some felt lucky to receive tailored support from charities and general practitioners. Many highlighted the need for improvements in integrated care frameworks for partners. These findings call for more inclusive and consistent care frameworks that actively involve, inform, and support partners with recognising and responding to symptoms of PP, involvement in care and supporting their wellbeing throughout. Further efforts are required to raise awareness of PP, improve staff training, and explore partners’ needs from varying backgrounds, time points, and circumstances.

## Introduction

Psychosis following childbirth, also known as postpartum psychosis (PP), affects approximately one to two per 1,000 women (Perry et al., 2021). Onset is often rapid and unexpected, with most cases emerging within two weeks postpartum (Heron et al., 2008). Symptoms include hallucinations, delusions, and disturbances in mood and cognition (Friedman et al., 2023). Symptoms are variable and change rapidly, with confusion and hypomania being common in the prodromal phase (Heron et al., 2008). Content of hallucinations and delusions is also variable but often relates to concerns and fears about the baby. Recognised as a ‘psychiatric emergency’, PP often results in psychiatric hospitalisation due to risk to the mother and baby (Heron et al., 2008; Jones et al., 2014). Despite its severity, PP is not classified as a distinct diagnosis in the DSM-5 (American Psychiatric Association, 2013) or ICD-11 (World Health Organization, 2019), instead, both include a specifier for ‘postpartum onset’ within four to six weeks after childbirth. Timely detection and treatment are crucial for a good prognosis, with early intervention leading to more favourable outcomes (Boyce & Barriball, 2010; Burgerhout et al., 2017; Jairaj et al., 2023). PP affects women from diverse backgrounds, with risk factors including pre-existing mental health conditions (namely bipolar disorder), previous PP episodes, obstetric complications, psychosocial stressors, immunological and genetic factors, and sleep deprivation (Işik, 2018; Jones et al., 2014; Perry et al., 2021). Experiencing PP can be life-changing and traumatic (Forde et al., 2020; Fusar-Poli et al., 2025; Heron et al., 2012), sometimes affecting maternal caregiving and bonding (Biaggi et al., 2021), which may affect child development (Plunkett et al., 2017). There is also an increased risk for maternal suicide, infant related accidents, and future psychiatric episodes (Brockington, 2017; Gilden et al., 2020).

Partners of women with PP often take on multiple responsibilities and roles as family dynamics shift, whilst also facing significant emotional and psychological challenges in navigating the condition (Doucet et al., 2012; Engqvist & Nilsson, 2011; Forde et al., 2020; Heron et al., 2012; Holford et al., 2018). PP can place strain on relationships (Wass et al., 2024), with research indicating that one in five marriages end following a PP episode (Blackmore et al., 2013). Conversely, some partners report that the experience of PP fosters greater understanding and support, leading to growth and positive change in their relationship (Boddy et al., 2017). Whilst partners often play a pivotal role in women’s postnatal care and recovery (Dolman et al., 2013; Grube, 2004; Jairaj et al., 2023; McGrath et al., 2013; Roxburgh et al., 2023), many express being excluded from care processes. They often feel uninformed about their partner’s diagnosis, status, care plans, and prognosis (Boddy et al., 2017; Doucet et al., 2012; Holford et al., 2018). Specialist services such as Mother and Baby Units (MBUs) are perceived as more inclusive and responsive than general acute psychiatric wards and community care (Boddy et al., 2017; Roxburgh et al., 2023). Partners also urge for practical support, such as help with household tasks and infant care, although this has rarely been offered (Boddy et al., 2017; Doucet et al., 2012). It is, however, important to note that pre-existing relationship difficulties and conflict with the partner may pose additional barriers and or worsen perinatal mental health outcomes (Antoniou et al., 2021; Howard et al., 2013).

The NHS Long Term Plan (NHS England, 2019), building on the Five Year Forward View (NHS England, 2016), states that by 2023/24, partners of women accessing specialist perinatal mental health services should receive mental health checks, be incorporated into evidence-based psychological therapies, and have signposting to support as needed. Likewise, the National Institute for Health and Care Excellence (NICE, 2020) guidance outlines the need for healthcare professionals to consider the role of the partner in care plans, provide them with information and advice, and assess the impact of PP on the couple’s relationship. However, many partners still lack access to the required specialist support, highlighting a critical gap between policies, guidelines, and service delivery. Ongoing work and resources to deliver targets set by the Long Term Plan are required to ensure this much needed care is provided (Maternal Mental Health Alliance, 2023; NHS England, 2021).

Although existing research provides valuable insights into the experiences of services among partners of women with PP, these studies were conducted before the implementation of the Long Term Plan; we would expect changes in service provision and inclusion of partners to have improved since then. To our knowledge, no previous studies have solely explored in-depth partners’ experiences of current service systems within the context of PP.

In the current study we aim to address these gaps in the literature by exploring how partners of women with PP experience mental health services, and what kind of care they would prefer. Gaining a deeper understanding of these experiences can further inform service provision for partners of women with PP, helping tailor care and enhance outcomes.

## Methods

### Design

This study adopted a qualitative design. Data were analysed using thematic analysis, following an inductive approach to identify commonalities and variations across transcripts (Braun & Clarke, 2006, 2019). This method was deemed appropriate given the exploratory nature of the research, facilitating a comprehensive analysis that extended beyond individual accounts to uncover patterns across the broader dataset (Braun & Clarke, 2006). A critical realist and contextualistic epistemological and ontological stance was assumed, recognising individuals’ experiences as authentic yet shaped by their contexts, thereby producing knowledge that can inform meaningful change (Rogers & Rogers, 1997; Tebes, 2005). NHS Health Research Authority approval for the study was obtained from South Central Oxford B Research Ethics Committee (reference: 24/SC/0013).

### Participants

Twelve partners of women who all self-reported having experienced PP were included in this study. Partners were purposively recruited through three charities (Action on Postpartum Psychosis [APP], Bipolar UK, and the Home-Start project, Dad Matters) and one NHS healthcare provider in the East of England to ensure diversity in demographic and socioeconomic factors, as well as service use. Inclusion criteria required partners to be 18 years or older; be the partner of a woman who received care for psychosis following childbirth (self-reported as PP by women) in the UK; and to have been in a relationship with the woman at the time of birth and throughout the postnatal period. Whilst partners views following relationship breakdown are important, it was agreed that this criterion would provide the most homogenous and comparable insights. The PP episode must have occurred within the past seven years and more than six months ago. In line with previous studies (Doucet et al., 2012; Grube, 2004; Holford et al., 2018), a time frame of seven years was chosen to balance event recall with ensuring an adequate sample size given PP’s low prevalence (Perry et al., 2021). This time frame also ensured that support was accessed following the release of the UK 2016 Five-Year Forward View (NHS England, 2016). A six-month minimum since onset aimed to reduce distress and ensure partners had sufficient service experience and time for reflection. Partners who did not understand or speak fluent English were excluded. Participants meeting inclusion criteria who expressed an interest in participating were contacted by the first author to provide them with more information about the study, review informed consent, and schedule the interview. Participants were informed that their contributions would be kept confidential, with anonymity of quotations ensured. An incentive of £10 in the form of a voucher was offered.

### Data Collection

Recruitment involved online and in-person leaflet advertisement directing interested individuals to an online screening questionnaire. This questionnaire collected contact details, verified inclusion criteria, informed participants about the research, and obtained informed consent. Interviews were semi-structured, using an interview schedule containing open questions to guide areas of discussion. This explored partners’ wellbeing, and their views of the services accessed for the woman’s PP throughout her entire care pathway. Characteristics pertaining to the participant and their partner (reported by participant) were obtained at interview stage. Interviews were conducted via Microsoft Teams between March and July 2024 and lasted approximately one hour and 30 min. The interview schedule was informed by existing research (e.g., Holford et al., 2018; Roxburgh et al., 2023) and developed by the research team. The interview scheduled, coupled with the advertisement of the study, were reviewed by two partners of women who experienced perinatal mental health difficulties, with suggestions for refinement actioned. Interviews (*n* = 12) were conducted by the first author, a clinical psychology doctoral trainee who had received training in qualitative research methods and clinical skills for engaging with potentially vulnerable groups.

### Analysis

Interviews were video recorded and transcribed verbatim by the first author (ER), which aided with data familiarisation (Riessman, 1993). Identifying features reported during interviews were anonymised. Interviews were then imported into NVivo 14 for analysis by ER. Thematic analysis (Braun & Clarke, 2006, 2019) was adopted, taking an inductive approach exploring meaningful comments and patterns in partners’ experiences. Transcripts were read and re-read to identify initial codes which were then developed into themes. Themes were refined and restructured as the analysis progressed. To enhance rigour, a second researcher (JH) reviewed initial codes and read a subsample (*n* = 3) of interviews. Regular discussions were held among the research team throughout the analytic process to revisit and adjust the thematic framework. Given the emphasis on reflexivity in qualitative research (Dodgson, 2019), the researchers regularly considered how their beliefs, biases, and positions might influence the data interpretation and theme development.

## Findings

### Participant Characteristics

Participants’ characteristics are shown in Table 1, with additional information regarding their partners where relevant. Their mean age was 39 (range 31 to 45). Most participants were highly educated, full time employed, and from a white British background. All participants identified as male, were married, and living with their partner. There was variability in the mental health services accessed by women for their PP support, which is not uncommon (see Table 1). Of the 12 women, two did not access specialist perinatal support at any point. Table 2 provides details of the key services that participants reported women accessing. Some women were reported to have engaged with more than one service at the same time during different stages of their care journey.

**Table 1:** Characteristics of participants and their partners (*N* = 12)

Characteristics		Category	Mean or *n*
Participant’s age (years)		Mean Age	39
Gender		Male	12
Ethnicity		White British	8
		White Other	3
		Asian	1
Level of education		Postgraduate degree or above	8
		Undergraduate degree	2
		A-Levels	1
		GCSE	1
Employment status		Full time employed	9
		Part time/self-employed	3
Marital status		Married	12
Living with partner		Yes	12
Number of children		1	4
		2	6
		3+	2
Pre-existing mental health status in participant (self-reported)		Anxiety	3
		No prior experience	9
Psychiatric status in women (reported by participants)		First onset of postpartum psychosis (one woman with pre-existing schizophrenia)	10
		Second onset of postpartum psychosis	2
Services accessed in postnatal period by women	Specialist perinatal	Mother and baby unit	7
(women could access more than one service)		Perinatal community mental health team	8
	Non-perinatal	General acute psychiatric ward/psychiatric intensive care unit	5
		General community mental health team	4
		Early intervention in psychosis services	4
		Crisis resolution team/home treatment team	7
Time since support accessed for first PP episode		1 year or less	4
		2–4 years	5
		5–7 years	3

**Table 2:** Details of mental health services accessed by women

Specialist perinatal or non-perinatal service	Type of service	Description
Specialist perinatal	Mother and baby unit	Specialist inpatient unit supporting women with severe mental health needs and their babies.
	Perinatal community mental health team	Multidisciplinary team supporting women in the community with severe mental health needs.
Non-perinatal	General acute psychiatric ward/psychiatric intensive care unit	Inpatient wards for adults with severe mental health needs/providing higher security and intensive care. Women are admitted without their baby.
	General community mental health team	Multidisciplinary team supporting adults in the community with ongoing mental health difficulties.
	Early intervention in psychosis services	Multidisciplinary team supporting adults with a first episode of psychosis.
	Crisis resolution team/home treatment team	Multidisciplinary team supporting adults in an acute mental health crisis.

### Overview of Qualitative Findings

Three main themes were identified from the analysis: ‘understanding and responding to postpartum psychosis’, ‘the responsibilities undertaken and partner wellbeing’, and ‘partners’ involvement in postpartum psychosis care’. Table 3 shows subthemes for these, each described in detail in the text below.

**Table 3:** Themes and subthemes identified

Themes	Subthemes
Understanding and responding to postpartum psychosis	Recognising the early warning signs Staff awareness and communication Bridging the knowledge gap
Partners’ involvement in postpartum psychosis care	Using their voice Partners in the picture Improving inclusive care frameworks
The responsibilities undertaken and partner wellbeing	Carrying the load The emotional toll on partners

### Understanding and Responding To Postpartum Psychosis

Many participants struggled to recognise their partner’s mental health decline, often realising its severity when symptoms became extreme. Variability in healthcare professionals’ knowledge of PP contributed to their frustration and confusion, further diminishing participants’ confidence in the system. Many stressed the need for greater awareness and accessible information about PP, not only for partners and healthcare professionals, but also wider society.

#### Recognising the Early Warning Signs

Recognising the early signs of PP proved difficult for many participants, as they struggled to understand the changes in their partner’s mental health presentation. Some misattributed symptoms to physical health complications or medication side effects. Several participants only realised the severity of the situation once their partner reached crisis point.

She was definitely low, getting worried, and her behaviours were becoming strange…it got to the point where I’d woken up one morning and she was wide awake, and I just turned around and said to (her) you ok? And she said, yeah we’re dead, I think we’re dead, and (name of child)…we are dead. And I think that’s when I realised something was definitely not right. It wasn’t just side effects of the tablets. (P09)

This lack of awareness often delayed help-seeking, leaving some questioning whether it had worsened their partner’s prognosis. A few participants felt that their professional experiences had equipped them to recognise their partner’s mental health decline and take appropriate action, noting that ‘that’s probably what made the difference’ to their partner’s prognosis (P03). Midwives often facilitated access to support services, whilst participants without such guidance relied on general practitioners (GPs) or attended Accident and Emergency (A&E), unaware of the support options available. In contrast, one woman’s pre-existing schizophrenia diagnosis ensured she was already under the care of services. Participants vividly recalled these early events, reflecting confusion, urgency, and, at times, regret over delayed action.

It was like in a movie…on my way home, I didn’t really know what was going to be there. And I was worried that the children will be dead, like literally, in the washing machine or something because I knew that she was, she wouldn’t even recognise me over the phone. (P03)

#### Staff Awareness and Communication

Participants described disparities in healthcare professionals’ awareness and communication of information regarding PP from conception through to postnatal care. Several were surprised by the lack or absence of information on severe mental health conditions during antenatal and National Childbirth Trust (NCT) classes, describing these as missed opportunities to raise awareness.

…they could have flagged it up a bit more the kind of mental health impacts that having a baby can have… that there’s potential that you can experience a serious mental illness…it would be good to know that that’s a possibility…I would have just like to have been a lot more alert to the possibility that this could take a real toll on my wife’s mental health and that it’s not just stress. (P12)

Many felt healthcare professionals lacked expertise in recognising PP, with some highlighting instances when their partner was misdiagnosed with postnatal depression (PND). Of the 12 women, eight were told by healthcare professionals that they were experiencing PP, although this information was sometimes not relayed to participants. One woman was told that she was exhibiting PP symptoms, but this was never officially confirmed. Whilst another woman’s symptoms were managed as a relapse of her pre-existing schizophrenia, despite the participant’s efforts to highlight how different this episode felt in comparison to previous relapses. Two were later diagnosed with bipolar disorder, with murmurings of PP-like symptoms by healthcare professionals earlier in the woman’s care. The lack of clarity regarding their partner’s presentation added to participants’ confusion, with some attributing this to perceived delays in appropriate care.

I think that they weren’t aware of the severity of the situation. I certainly wasn’t, and I think them as the professionals, albeit midwives, weren’t aware, or at least didn’t have enough awareness. I know they’re not mind readers…but I think that perhaps if they’d had more…of an awareness, they could have given me more of an awareness. (P06)

Many emphasised the importance of reassurance about the prognosis of PP, which few felt was adequately provided. Some sought information online or via charities like APP or Bipolar UK, often on their own initiative due to limited signposting from services.

#### Bridging the Knowledge Gap

Many participants lacked prior knowledge of maternal mental health conditions, with nearly all having not heard of PP before. They emphasised the need for greater awareness not only among healthcare professionals but also for partners and wider society. Suggestions included providing practical resources, such as leaflets or emails directed at partners, with clear information about warning signs, potential presentations of PP (and other conditions), and support systems available.

I expressed this in many moments of frustration, even if the staff are not aware in detail of the intricacies of mental health challenges around perinatal period, surely at the very least there could just be a pack that gets given to every expected mother to take home to share with their partners that has, you know, here’s the links to the websites, here’s the phone numbers for perinatal teams if you need to contact them…it gives them a point to try and start accessing the system. (P08)

Participants believed having this information pre and post birth could have made them more alert and responsive to their partner’s mental health needs. However, some acknowledged that these materials might not have always been read and retained.

### Partners’ Involvement in Postpartum Psychosis Care

Participants provided unique insights into their partner’s condition and often advocated on her behalf when she could not due to the severity of her PP symptoms. They experienced varied levels of involvement from services, highlighting the need for better integrated care frameworks to support their active role.

#### Using their Voice

Participants were pivotal in both advocating for their wife’s needs and taking initiative to ensure care progressed smoothly. For example, they ensured services followed previously established care plans or requested changes to new ones and arranged appointments and care in the initial stages and during relapses.

She (healthcare professional from crisis team) said…I got your wife booked for Tuesday. And I said, no…this is something that needs to be treated for immediately, so I would really appreciate if she was seen today. And actually the same day at around 12, we had…a psychiatric consultant and mental health nurse coming in. (P03)

One participant took the lead in securing an ambulance to transport his partner to an MBU. Several participants also led discussions with healthcare professionals when their partner’s PP symptoms were severe. Whilst attending services, partners often served as emotional anchors, stepping in to support their partner during acute episodes. Despite their efforts, some partners hesitated to speak up, feeling unsure of their role or deferring to healthcare professionals. Familiarity with the system helped some gain confidence to advocate more effectively.

I’ve learned so much more about how you…navigate the system…which leavers to pull and buttons to push to get the right outcomes…hopefully…this conversation is part of a drive to make it so that people don’t have to learn it the hard way as much. (P08)

#### Partners in the Picture

The involvement of participants in PP care varied greatly across services and individual experiences. Participants described feeling valued and included in many instances but ignored and overlooked in others, with unclear reportings of either more or less favourable outcomes in more recent years.

…she was like and what is (wife) like normally?…I hadn’t really expected it based on the experiences I’d had up to that point for me to be asked anything other than when does (baby) need feeding…so that was good. (P01, MBU)

Several participants noted missed opportunities for updates, invitations to discharge planning or care meetings, and inclusion in care decisions. Whilst COVID-19 posed challenges, some felt it did not justify the lack of involvement or support they experienced. Participants’ involvement was sometimes facilitated by their partner requesting their presence. In other cases, they felt PP symptoms caused their partner to doubt their intentions.

I’ve been a little worried to contact the MBU because I don’t want the same thing to repeat again, like my wife accusing me of having an affair with the staff members. (P11)

Many participants spoke positively about MBUs and acute wards for acknowledging and promoting family involvement, although others had less favourable experiences. Meanwhile, perinatal teams were praised for their support but also criticised for their shortcomings, as participants sometimes felt ‘on the outside’ of care. This was also the case for crisis and general community mental health teams, where participants reported positive experiences of feeling seen and heard, with other reports of being kept in the dark. These varied experiences highlight potential inconsistencies in how participants have been involved in PP care, even when supported by the same type of service.

#### Improving Inclusive Care Frameworks

Participants highlighted the need for improved care frameworks that actively involve them not only throughout the PP care process but also from conception.

…not living in like the old age times where the mums are doing everything type thing…just being involved…or like being looked at when those discussions are happening rather than feeling like you, you’re sort of sat on the side or in the corner or going out to do the tea or coffee run. (P01)

Whilst two participants expressed appreciation for the NHS and access to specialist support, others shared concerns with the system, such as feeling like services were designed only with women in mind, healthcare professionals lacking knowledge of women’s mental health history, and disorganisation and delays in obtaining transport to inpatient facilitates. Staffing issues and confidentiality policies were cited as barriers to accessing information. There were also calls for better communication between and within services, alongside improved community care models for including women’s partners.

…perhaps pick up some points from the MBU…because that…was 10 out of 10…and putting that into the community model…would be really, really, really helpful…it would make the recovery better for mum…and make the whole process more efficient and reassure dad as well. (P06)

Participants also emphasised the need for services to acknowledge their role and proactively involve them by providing information, signposting resources, and actively listening to their concerns.

…it would be more helpful if the onus was actually on the doctors and nurses to call you, to give you the updates rather than you to be chasing them, probably a couple of reasons for that, one of them…there comes a point when you’re like, I’m just harassing them, and they’ve got more to do than just speak to me and I got to the point where I realised I wasn’t going to get much out of calling them, so I just stopped calling them. (P09)

### The Responsibilities Undertaken and Partner Wellbeing

Most participants faced diverse and overwhelming responsibilities when their partner was unwell, sometimes shaped by systemic factors. These demands, coupled with the experience of PP, often affected their wellbeing, with some feeling supported whilst others felt left to manage alone.

#### Carrying the Load

It was common for participants to take on significant responsibilities whilst managing their partner’s PP. Many assumed childcare duties, especially when their partner was not admitted to an MBU, often relying on family or friends for essential support.

It was a logistical nightmare…my mum’s only a 10 min walk down the road now ‘cause she moved to help with everything that happened. (P06)

Participants also managed feeding, household chores, picking up prescriptions, finances, and implementing care advice from community teams to ensure the safety of their partner and child(ren). They were also sometimes required to step into the role of decision-maker on behalf of their partner. Long distance travel to inpatient wards or MBUs added to the strain. Some participants sought childcare solutions to continue working, although this could be challenging, whilst others found work to be an escape.

I’m in a situation where my wife is in the adult psychiatric ward. I have a 5 month baby to look after. I’m unable to go to work. I don’t have any childcare in place. It’s way too expensive…I requested social services to help me…I’m in a situation neither I’m able to go to work, neither am I getting childcare, neither am I getting an MBU…I had a breakdown, what do I do? (P11)

#### The Emotional Toll on Partners

The experience of PP had a profound emotional impact on many participants, sometimes resulting in mental health difficulties such as depression, anxiety, or PTSD.

…there are lots of things that my wife doesn’t remember about everything that happened….whereas I can’t forget any of it. I saw it all…there’s no forgetting for us. (P05)

The strain also affected relationships, with couples therapy often proving beneficial but typically sought privately rather than signposted by services. Many participants adopted a ‘head down, just get on with it’ mentality, immersing themselves in supporting their partner and managing responsibilities.

…bit of a typical bloke…I also feel like I just need to crack on…there’s stuff that I need to deal with here, and yeah I feel a bit down, but it’ll pass and we’ll just crack on and get the job done. (P04)

Whilst this approach helped them cope, some felt it reinforced the ‘stereotypical man’ image, which some thought led services to assume they were coping. Participants frequently described feeling overlooked by services, with few having received check-ins, signposting, or support for their wellbeing. Barriers such as time-limited GP appointments, online referrals, and uncertainty about where to access support hindered help-seeking. Many were frustrated with services’ inadequate signposting and advertisement of APP, which was often the only source of support accessed by participants. Some accessed other charities like Dad Matters and Bipolar UK, received support via their employer, or self-referred to talking therapy services. Two participants praised their GP’s for proactively and consistently checking in on their wellbeing. Delayed mental health declines commonly emerged after their wife stabilised, during subsequent pregnancies, or when no further episodes occurred.

…they said everything was absolutely fine (wife’s mental health after second pregnancy) and I went into the garden and sobbed…I just realised how much I had been gearing myself up for this next fearful fight. (P02)

Participants tended to downplay their struggles but urged services to be more direct with check-ins and to provide hope during PP episodes.

## Discussion

This study aimed to build upon existing research by exploring in-depth partners’ experiences of current service systems following the implementation of the Long Term Plan within the context of PP care, whilst also focusing on better understanding what kind of support they would prefer. In line with previous research (Boddy et al., 2017; Engqvist & Nilsson, 2013; Forde et al., 2019), partners struggled to recognise and make sense of their wife’s mental health decline, with some attributing changes in mood to physical health complications post birth. This occasionally delayed help-seeking, which is particularly problematic in the context of PP, given the immediate need for intervention to mitigate risks and improve outcomes (Boyce & Barriball, 2010; Burgerhout et al., 2017). Symptoms of PP can further hinder women’s abilities to seek support themselves, placing greater reliance on partners (Roxburgh et al., 2023).

Aside from their own lack of awareness of PP, partners reported perceived disparities in healthcare professionals’ knowledge of the condition across the perinatal period, comparable to existing findings (Lyons et al., 2024). This sometimes led to misdiagnosing PP for PND (Holford et al., 2018), further exacerbating existing confusion among partners. Heterogeneity of PP poses significant diagnostic challenges (Sharma et al., 2022), potentially hindering healthcare professionals’ confidence in detecting, responding to, and informing partners about the condition. Calls for greater societal awareness regarding PP were prevalent among partners, something that organisations like APP are working hard to address.

Partners can play a critical role throughout the journey of PP, providing essential support, advocating during women’s care, and offering stability. Contrary to prior research (Boddy et al., 2017; Doucet et al., 2012; Lever Taylor et al., 2018; Holford et al., 2018), many partners felt that services embraced their role, reflecting progress towards the NHS Long Term Plan’s (NHS England, 2019) objectives to enhance partner involvement in perinatal mental health care by 2023/24. However, systemic barriers to care and variability in involvement across services and individual experiences remained, indicating the need for further efforts to ensure these frameworks are consistently and effectively implemented across all settings.

Additionally, it is important to recognise the role either PP related symptoms (such as delusions and paranoia) and or pre-existing difficulties within romantic relationships (such as mistrust, lack of support, abuse) may pose for partner involvement. As reported within this study and pre-existing research (Forde et al., 2020), women may become more sceptical of their partner’s intentions during their care, a key differentiating factor between PP and other perinatal mental health conditions when considering tailored support (Fusar-Poli et al., 2025; O’Hara & Wisner, 2014). Ensuring adequate information gathering throughout antenatal care (e.g., routine midwife appointments) could prove vital in recognising any potential strains within the relationship. Likewise, decisions regarding partner involvement should not be static, given the variability of a PP presentation. Thus, embracing a dyadic way of working and reassessing partner involvement at different points in the woman’s care could be helpful. Partners stressed the importance of inclusive care frameworks that acknowledge their role from conception through recovery, aligning with guidelines proposed by NICE (2020) and NHS England (2021). Similarly, as highlighted in a qualitative systematic review conducted by Lyons et al. (2024), the need for healthcare professionals to provide reassurance and information on PP was reiterated by several partners.

Consistent with previous research (Boddy et al., 2017; Doucet et al., 2012; Heron et al., 2012; Holford et al., 2018), the significant responsibilities placed on partners often left them feeling overwhelmed, with many adopting a ‘just get on with it’ mentality to cope. Whilst this approach appeared to help them manage immediate demands, it may have masked the emotional toll of their experiences, with mental health challenges often emerging once things seemed to settle (in line with previous research by Heron et al., 2012). This period is also accompanied by partners attempting to rebuild trust in their relationship after witnessing distressing events during the acute phase of PP (Boddy et al., 2017; Holford et al., 2018; Wyatt et al., 2015). Partners also reported difficulty expressing their struggles to services, sometimes hindered by systemic factors such as complicated referral processes and time-limited appointments. It is important that healthcare professionals remain alert to these dynamics.

Women who experienced PP have recognised the psychological impact it can have on their partner and their need for psychological support (Heron et al., 2012; Roxburgh et al., 2023). However, repeated gaps in services’ recognition of and offerings of support for partners have been highlighted (Doucet et al., 2012; Holford et al., 2018; Roxburgh et al., 2023), findings echoed in this study. A recent Maternal Mental Health Alliance (2023) report further underscores this, noting that as of 2023, only 42% of services offered mental health assessments and signposting for partners. Therefore, there remains an urgent need for more consistent and proactive approaches within services to ensure healthcare professionals are effectively identifying and supporting the mental health needs of partners. There also needs to be greater attention paid to addressing the strain that PP places on relationships, with services signposting to relevant support rather than leaving it to be sought privately, as noted by partners in this study.

### Limitations

Whilst this study provides unique insights into partners’ experiences of care during PP, several limitations must be acknowledged. Data analysis was conducted by the first author, but regular discussions with the research team ensured a triangulation approach to enhance rigour. Additionally, despite efforts made to mitigate recall bias, the time elapsed and distress caused by the episode may have influenced participants’ recall. The sample consisted predominantly of white British, highly educated, married male partners, thus limiting the transferability of findings to partners from other backgrounds, gender identities, or relationship status. Findings are also limited to those who have not experienced relationship dissolution following a PP episode. Likewise, as participants were recruited predominantly through charities, they may represent a more proactive group and not speak to the views of those unable to access such support. As partners accessed services across the UK, findings are likely to be most transferable to these settings, although they may hold relevance for international contexts. Lastly, half the sample experienced PP during the COVID-19 pandemic. Whilst some partners felt the pandemic did not influence their experiences, the extent to which service pressures impacted care remains unclear.

### Implications

Providing partners with accessible and tailored information regarding maternal mental health, including PP where appropriate, during antenatal (e.g., midwife appointments and NCT classes) and postnatal care is an important consideration for improving their awareness and preparedness. Also, ensuring standard information gathering is undertaken during antenatal appointments to better understand relationship dynamics. Midwives are well positioned to facilitate these discussions due to their regular contact with families throughout the perinatal period. They would also benefit from further training to build their confidence in recognising and responding to PP. Likewise, despite classification complexities regarding PP, training could also be extended to healthcare professionals across all perinatal care disciplines to enhance consistent recognition and management of PP. This would not only improve timely and appropriate care for women but also provide clarity and reassurance to partners.

Greater societal awareness of PP is required, and although charities like APP have made significant progress in this area, continued efforts are required to support earlier recognition of symptoms and encourage timely help-seeking. As outlined in NHS England’s (2021) guidance, the necessity to ‘think family’, ‘remain curious’, and maintain a ‘perinatal frame of mind’ is underscored by partners in this study, reiterating further efforts required to ensure the smooth running of said principles in current care frameworks. Likewise, services need to ensure that confidentiality policies do not inadvertently exclude partners, and that care adopts a family centred approach as recommended by NICE (2020). Due to partners known delayed mental health decline and help seeking, further efforts should be made to proactively offer mental health assessments, tailored interventions, and signposting to relevant organisations (e.g., APP) at various time points.

Given the predominantly white British, highly educated partners in this study, future research would benefit from exploring the experiences of partners from diverse sociocultural and economic backgrounds, whose perspectives remain underrepresented. This is particularly pertinent as partner characteristics in this study (e.g., stronger social set ups) may have offered protective effects (Roxburgh et al., 2023), raising the question of whether partners from different backgrounds might experience greater challenges. Likewise, existing research highlights that ethnic minority women face significant barriers when accessing perinatal mental health support, such as stigma, language barriers, and a lack of culturally relevant information and care (Watson et al., 2019). Such insights could help tailor support to better address the needs of partners from diverse backgrounds.

Additionally, further research investigating the longer-term impacts of PP on partners is warranted, given the strain PP can place on relationships, the delayed mental health impact of these experiences, and uncertainties around future pregnancy, conception, and potential relapse. Likewise, understanding how partners manage the shift from the woman’s PP episode to an established diagnosis of bipolar disorder, particularly within the context of mental health services and the recognition of their caregiving role and needs, could offer valuable insights.

## Conclusions

This study explored partners’ experiences of mental health services in the context of PP, highlighting the roles they can play in caregiving, advocacy, and sustaining the family unit. Partners face significant emotional and practical strain, emphasising the need for services to proactively offer support and signposting for their wellbeing. Whilst some progress has been made, ensuring consistent involvement of partners across all settings remains essential. Greater awareness of PP among the public, healthcare professionals, and partners is necessary to ensure those involved are better informed and equipped to effectively manage PP episodes. Future research is needed to examine the experiences and needs of partners within the context of PP, particularly those from diverse backgrounds, differing relationship status, and at various stages of the caregiving journey.

## Data Availability

Data and materials are not available for distribution to others than the research team.
